# Supplementary material for: mTORC1 upregulates B7-H3/CD276 to inhibit antitumor T cells and drive tumor immune evasion
Source: Nat Commun. 2023 Mar 3;14:1214. doi: 10.1038/s41467-023-36881-7 (PMC9984496; doi:10.1038/s41467-023-36881-7)
Supplement: Supplementary file 1 — Supplementary Information [file 41467_2023_36881_MOESM1_ESM.pdf]

Supplementary Information for

mTORC1 upregulates B7-H3/CD276 to inhibit antitumor T cells and drive tumor immune evasion

Heng-Jia Liu*, Heng Du, Damir Khabibullin, Mahsa Zarei, Kevin Wei, Gordon J. Freeman, David J. Kwiatkowski, Elizabeth P. Henske*.

*Corresponding author. Email: hliu1@bwh.harvard.edu (HJ Liu) & ehenske@bwh.harvard.edu (EP Henske)

Supplementary Fig. 1

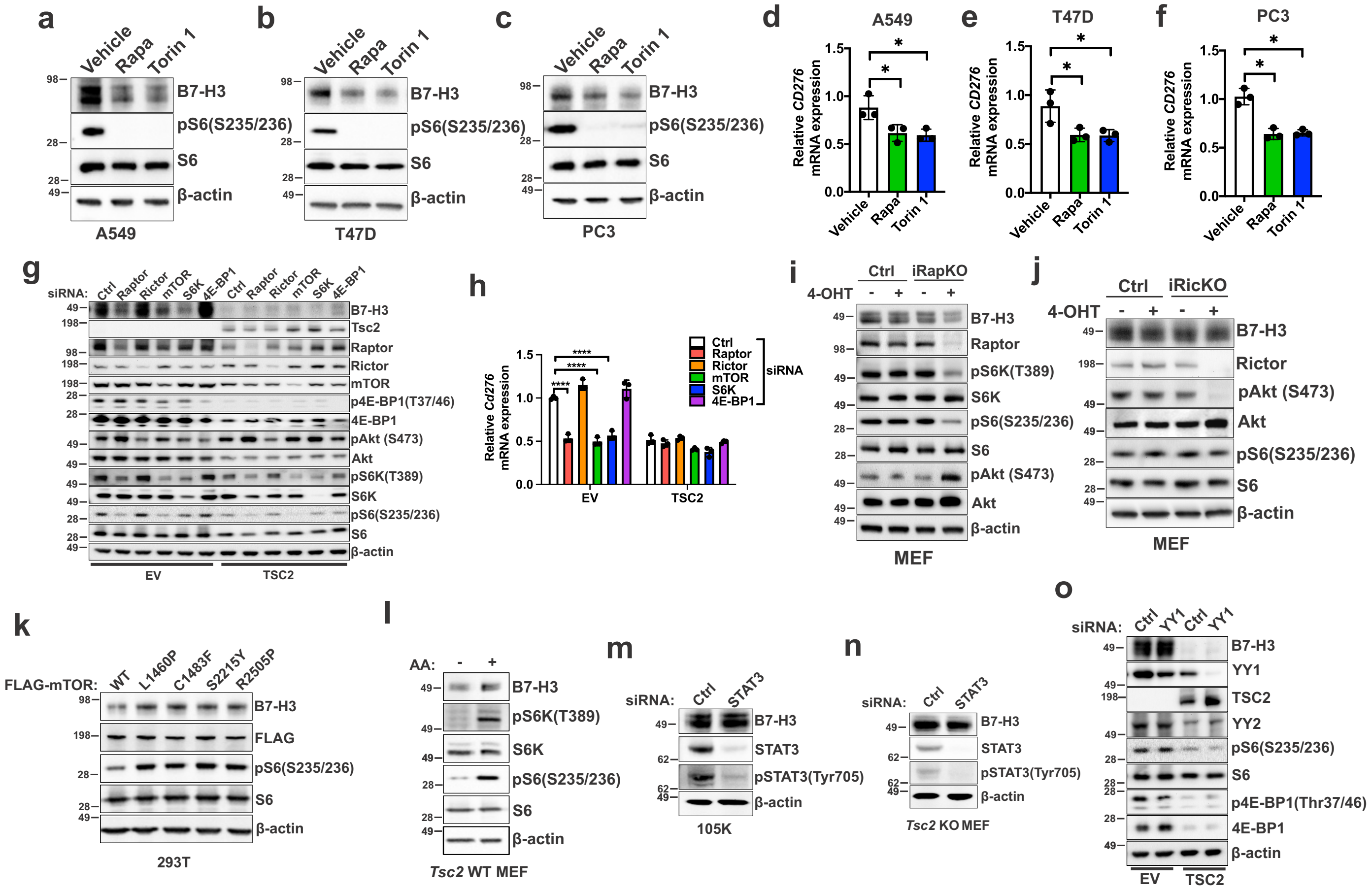

Supplementary Fig. 1. B7-H3 expression is regulated by mTORC1, but not mTORC2, STAT3 or YY1.

a-c, Suppression of B7-H3 protein expression in A549 cells **(a)**, T47D cells **(b)**, and PC3 cells **(c)** after treatment with Rapamycin (Rapa) or Torin 1 for 24 hrs (n = 3).

d-f, Suppression of B7-H3 mRNA expression in A549 cells **(d)**, T47D cells **(e)**, and PC3 cells **(f)** (n = 3) treated as in **a-c**. Means ± SD, one-way ANOVA with Dunnett's test, * $p < 0.05$.

g, Suppression of B7-H3 with downregulation of Raptor, mTOR, and S6K. *Tsc2-/-* 105K cells with stable reconstitution of TSC2 or empty vector (EV) were transfected with non-targeting control siRNA (Ctrl) or SMARTpool siRNA targeting Raptor, Rictor, mTOR, S6K, or 4E-BP1 for 48 hr (n = 3).

h, B7-H3 mRNA expression in the cells from **(g)** (n = 3). Means ± SD, two-way ANOVA with Holm-Sidak's multiple comparisons test, **** $p < 0.0001$.

i, Knockout of Raptor in iRapKO MEFs decreases B7-H3 expression. iRapKO or control (Ctrl) MEFs were treated with 1 μM 4-hydroxytamoxifen (4-OHT) for 72 hr (n = 3).

j, Knockout of Rictor in iRicKO MEFs does not affect B7-H3 expression. iRicKO or control (Ctrl) MEFs were treated with 1 μM 4-hydroxytamoxifen (4-OHT) for 72 hr (n = 3).

k, Immunoblot analysis of HEK293T cells transfected with wild-type mTOR (FLAG-mTOR-WT), constitutive active mutant mTOR in expression vectors followed by whole-cell-lysis 48 hr after transfection (n = 3).

l, Immunoblot analysis of WT MEFs that were starved of amino acid for 16 hr and re-stimulated with MEM amino acid for 24 hr (n = 3).

m,n, B7-H3 protein expression in *Tsc2-/-* 105K cells **(m)** and *Tsc2* KO MEFs **(n)** following transfection with control siRNA or SMARTpool siRNA targeting STAT3 for 48 hr (n = 3).

o, B7-H3 mRNA expression in *Tsc2-/-* 105K cells with stable reconstitution of TSC2 or empty vector (EV) following transfection with control siRNA (Ctrl) or SMARTpool siRNAs targeting YY1 for 48 hr (n = 3).

Source data and exact p values are provided in the Source data file.

Supplementary Fig. 2

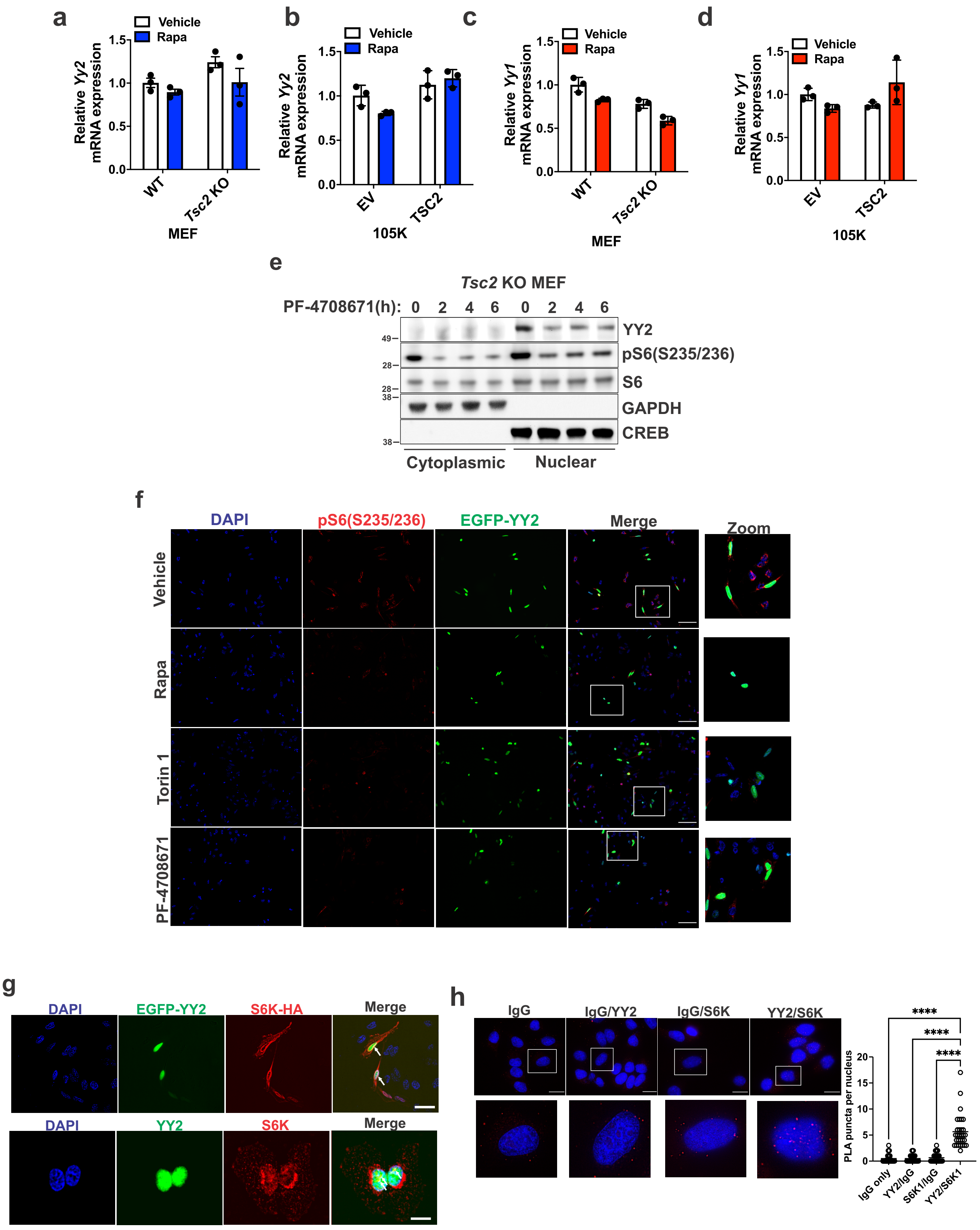

Supplementary Fig. 2. S6K forms a complex with YY2 in the nucleus.

a,b, YY2 mRNA expression in *Tsc2*-WT and *Tsc2* KO MEFs **(a)** and *Tsc2-/-* 105K cells with stable reconstitution of TSC2 or empty vector (EV) **(b)** following 20 nM Rapamycin (Rapa) or vehicle control for 24 hr (n = 3). Means ± SD, two-tailed unpaired student's t-test.

c,d, YY1 mRNA expression in *Tsc2*-WT and *Tsc2* KO MEFs **(c)** and *Tsc2-/-* 105K cells with stable reconstitution of TSC2 or empty vector (EV) **(d)** following 20 nM Rapamycin (Rapa) or vehicle control for 24 hr (n = 3). Means ± SD, two-tailed unpaired student's t-test.

e, Cytoplasmic and nuclear fractions of *Tsc2* KO MEFs following 10 µM PF-4708671 treatment for the indicated time points (n = 3).

f, Immunofluorescent staining of HeLa cells stably expressing EGFP-YY2 and treated with 20 nM rapamycin (Rapa), 500 nM Torin 1, 30 µM PF-4708671 or vehicle for 2 hr. Cells were fixed and stained with pS6 (S235/S236) and DAPI (n = 3). Scale bar = 100 µm.

g, Immunofluorescent staining of parental HeLa cells with anti-YY2 and S6K antibodies (n = 3). White arrows indicate co-localization of YY2 and S6K. Top panel: scale bar = 100 µm, bottom panel: scale bar = 20 µm.

h, Left panel: Representative images of in situ proximity ligation assay between S6K and YY2 (red) in HeLa cells. Data are representative of three independent experiments. Scale bar = 30 µm. Right panel: Quantification of the number of PLA puncta per nucleus. Column 1, IgG; column 2, mouse anti-YY2 and IgG; column 3, rabbit anti-S6K antibody and IgG; column 4, mouse anti-YY2 and rabbit anti-S6K antibodies. n = 30 cells. Means ± SD, one-way ANOVA with Dunnett's multiple comparisons test, **** $p < 0.0001$.

Source data are provided in the Source data file.

Supplementary Fig. 3

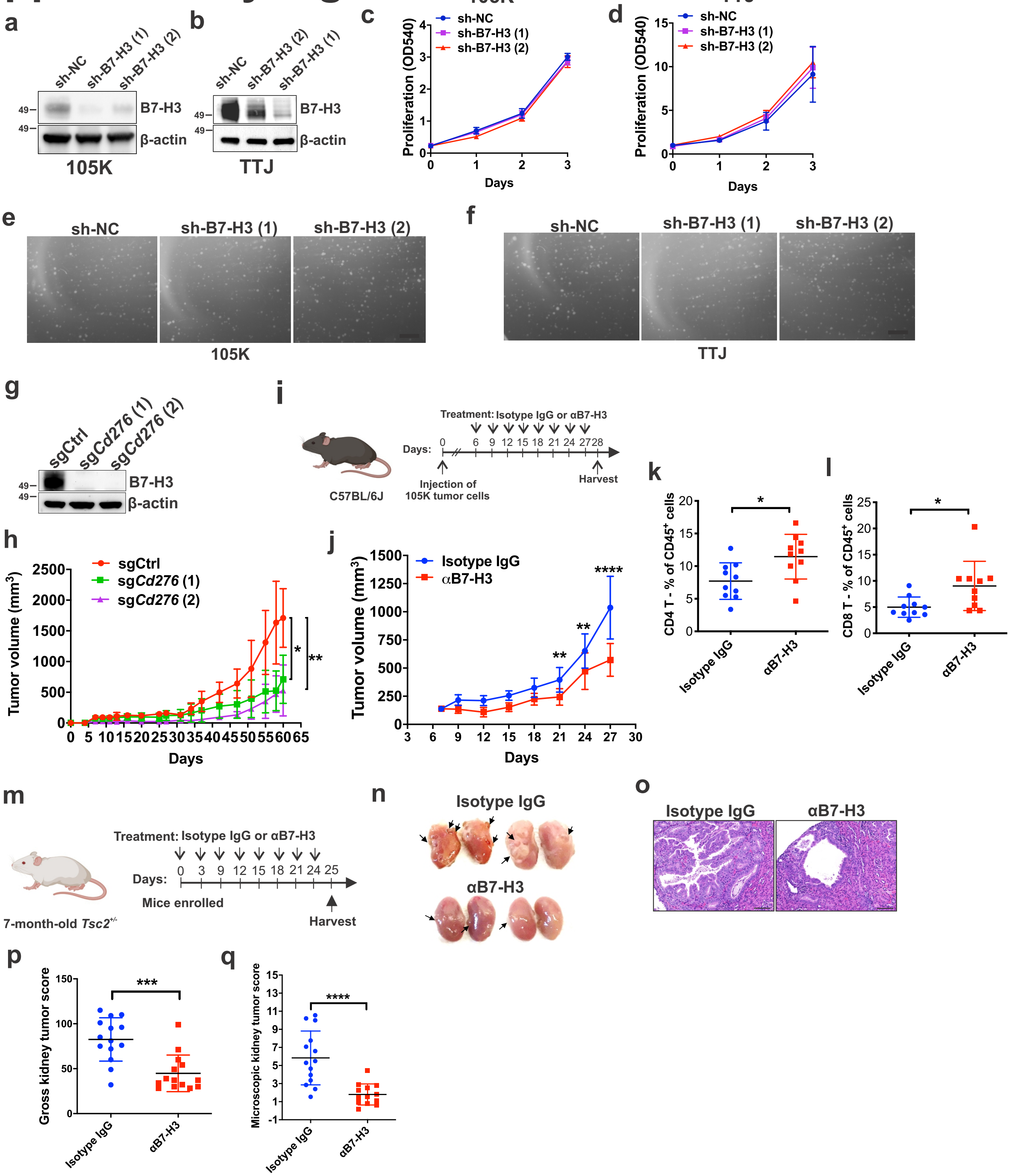

Supplementary Fig. 3. The effects of B7-H3 inhibition *in vitro* and *in vivo*.

a,b, Immunoblot of sh-NC, sh-B7-H3 (1), and sh-B7-H3 (2) *Tsc2-/-* 105K cells **(a)** or *Tsc2-/-* TTJ cells **(b)** (n = 3).

c,d, Cell proliferation assessed by crystal violet staining of the cells in **(c)** and **(d)** (n = 8). Means ± SD, one-way ANOVA with Dunnett's multiple comparisons test.

e,f, Representative images of the cells in **a, b** grown in soft agar for 3 weeks (n = 3).

g, Immunoblot analysis of whole-cell lysates derived from sgCtrl, sg*Cd276* (1), and sg*Cd276* (2) *Tsc2-/-* 105K cells (n = 3).

h, Subcutaneous growth of sgCtrl, sg*Cd276* (1), and sg*Cd276* (2) *Tsc2-/-* 105K cells in wild-type C57BL/6J mice. n = 7 mice for sgCtrl and sg*Cd276* (1), n = 8 mice for sg*Cd276* (2). Means ± SD, nonparametric Kruskal-Wallis's test with Dunn's multiple comparisons test, * $p < 0.05$, ** $p < 0.01$.

i, Experimental design for treatment of *Tsc2-/-* 105K tumors with isotype control antibody (Isotype IgG) or anti-B7-H3 antibody (α-B7-H3) (Created with Biorender.com).

j, Growth of *Tsc2-/-* 105K tumors treated as depicted in i. n = 9 mice for Isotype IgG, n = 10 mice for anti-B7-H3 antibody. Means ± SD, Mann-Whitney U Test, ** $p < 0.01$, **** $p < 0.0001$.

k,l, Flow cytometry analysis of tumor-infiltrating $CD4^+$ **(k)** and $CD8^+$ **(l)** T cells from *Tsc2-/-* 105K tumors treated as depicted in (I). 10 mice per treatment group. Means ± SD, Mann-Whitney U Test, * $p < 0.05$.

m, Experimental design for $Tsc2^{+/-}$ mice treated with isotype control antibody (Isotype IgG) or anti-B7-H3 antibody (α-B7-H3) (Created with Biorender.com).

n, Representative kidneys of $Tsc2^{+/-}$ mice treated as depicted in **m**.

o, Representative H&E images of $Tsc2^{+/-}$ mice treated as depicted in **m**.

p, Gross tumor score of kidneys of $Tsc2^{+/-}$ mice treated as depicted in **m**. n = 14 kidneys per treatment group, each dot represents one kidney. Means ± SD, Mann-Whitney U Test, *** $p < 0.001$.

q, Microscopic tumor score of $Tsc2^{+/-}$ mice treated as depicted in **m**. n = 14 kidneys per treatment group, each dot represents one kidney. Means ± SD, Mann-Whitney U Test, **** $p < 0.0001$.

Source data and exact p values are provided in the Source data file.

Supplementary Fig. 4

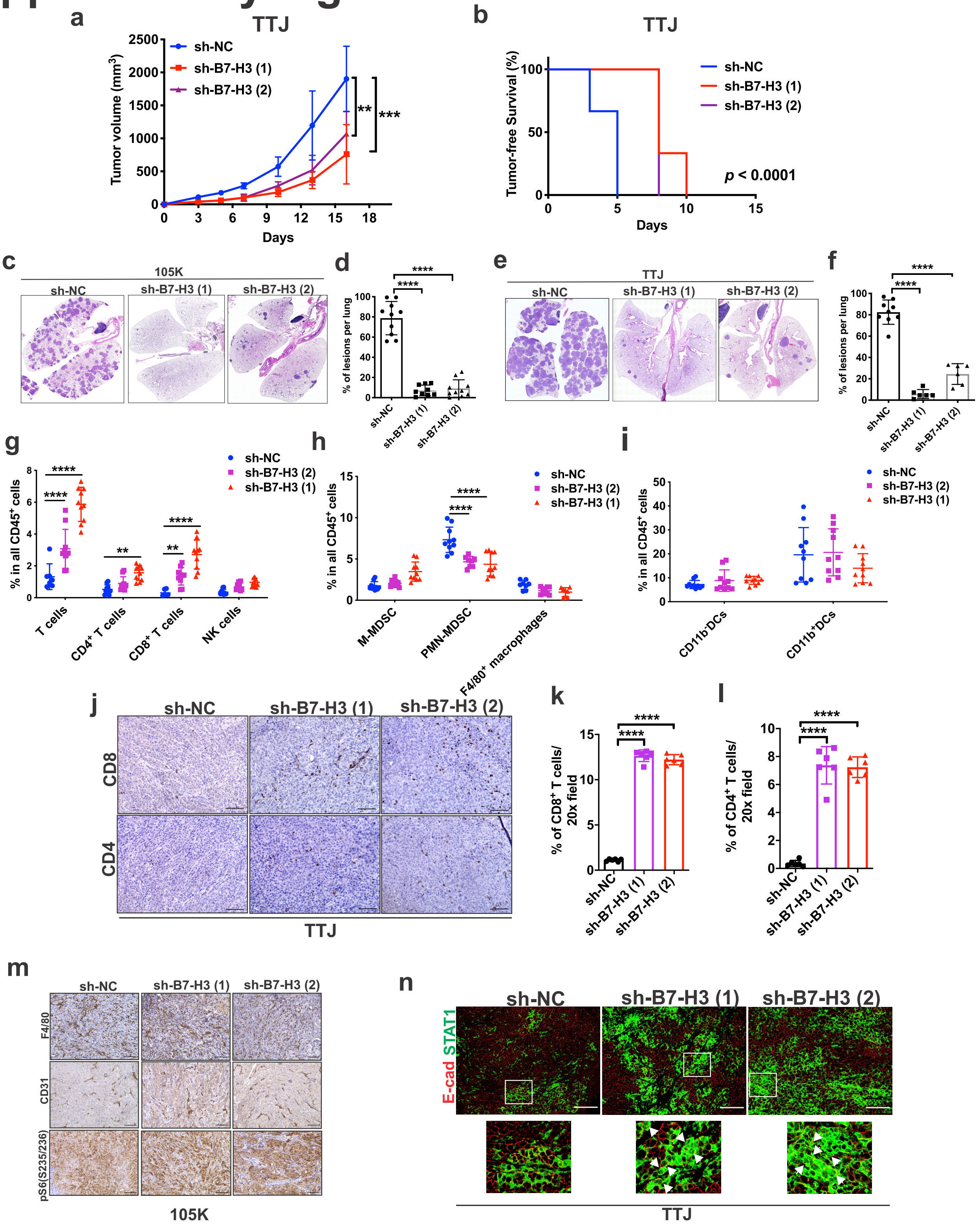

Supplementary Fig. 4. B7-H3 deficiency in *Tsc2-/-* TTJ tumor cells suppresses subcutaneous tumor growth and lung tumor burden in models of metastasis.

a, Subcutaneous tumor growth of sh-NC, sh-B7-H3 (1), and sh-B7-H3 (2) *Tsc2-/-* TTJ tumors in WT C57BL/6J mice. n = 6 mice per group, means ± SD, one-way ANOVA with Dunnett's multiple comparisons test, ** $p < 0.01$, *** $p < 0.001$.

b, Tumor-free survival curve of mice in **a**. Log-rank analysis.

c, Representative H&E images of lungs with sh-NC, sh-B7-H3 (1), and sh-B7-H3 (2) *Tsc2-/-* 105K tumors in WT C57BL/6J mice.

d, Percentage of lung area occupied by tumor per lung in **(c)**. n = 9 mice for sh-NC and sh-B7-H3 (2), n = 10 mice for sh-B7-H3 (1), means ± SD, one-way ANOVA with Dunnett's multiple comparisons test, **** $p < 0.0001$.

e, Representative H&E images of lungs with sh-NC, sh-B7-H3 (1), or sh-B7-H3 (2) *Tsc2-/-* TTJ tumors in WT C57BL/6J mice.

f, Percentage of lung area occupied by tumor per lung in (E). n = 6-9 mice per group, means ± SD, one-way ANOVA with Dunnett's multiple comparisons test, **** $p < 0.0001$.

g-i, Percentage of indicated cell types within $CD45^+$ TILs from sh-NC, sh-B7-H3 (1), and sh-B7-H3 (2) *Tsc2-/-* TTJ tumors. n = 7 tumors for T cells, n = 10 tumors for $CD4^+$ T and NK cells, n = 6 tumors for $CD8^+$ T cells in sh-NC, n = 10 tumors for all cell types in sh-B7-H3 (1), and sh-B7-H3 (2), means ± SD, two-way ANOVA with Holm-Sidak's multiple comparisons test, ** $p < 0.01$, **** $p < 0.0001$.

j-l, Representative images of CD4 and CD8 immunohistochemical staining on sections from *Tsc2-/-* sh-NC, sh-B7-H3 (1), and sh-B7-H3 (2) TTJ subcutaneous tumors **(j)**. Scale bar = 100 μm. Quantification of $CD8^+$ **(k)** or $CD4^+$ **(l)** T cells in each group (n = 6/group). Means ± SD, one-way ANOVA with Dunnett's multiple comparisons test, **** $p < 0.0001$.

m, Representative images of F4/80, CD31, and pS6 (S235/236) immunohistochemical staining on tumor sections from *Tsc2-/-* 105K subcutaneous tumors transduced with sh-NC, sh-B7-H3 (1), and sh-B7-H3 (2) (n = 3). Scale bar = 100 μm.

n, Representative images of E-cadherin and STAT1 immunofluorescent staining on subcutaneous TTJ tumor sections of the indicated groups (n = 3). Scale bar = 100 μm. White arrows indicate cells with nuclear STAT1.

Source data and exact p values are provided in the Source data file.

Supplementary Fig. 5

a

	Gene Signature	Size	ES	NES	NOM p-val	FDR q-val	FWER p-val	RANK AT MAX	LEADING EDGE
1	INTERFERON_ALPHA_RESPONSE	87	0.75	2.03	0.000	0.000	0.000	3098	tags=75%, list=18%, signal=90%
2	INTERFERON_GAMMA_RESPONSE	183	0.69	1.92	0.000	0.000	0.000	3098	tags=61%, list=18%, signal=74%
3	INFLAMMATORY_RESPONSE	181	0.6	1.69	0.000	0.002	0.007	3398	tags=45%, list=19%, signal=55%
4	ESTROGEN_RESPONSE_LATE	178	0.58	1.61	0.000	0.007	0.031	3317	tags=42%, list=19%, signal=51%
5	TNFA_SIGNALING_VIA_NFKB	194	0.56	1.57	0.000	0.009	0.051	3387	tags=44%, list=19%, signal=54%
6	ESTROGEN_RESPONSE_EARLY	186	0.55	1.55	0.000	0.010	0.070	3310	tags=39%, list=19%, signal=47%
7	IL6_JAK_STAT3_SIGNALING	83	0.57	1.53	0.002	0.013	0.099	2424	tags=39%, list=14%, signal=45%
8	ALLOGRAFT_REJECTION	171	0.53	1.48	0.000	0.019	0.161	3484	tags=36%, list=20%, signal=45%
9	KRAS_SIGNALING_UP	180	0.49	1.36	0.006	0.087	0.602	3630	tags=37%, list=21%, signal=46%
10	KRAS_SIGNALING_DN	144	0.47	1.32	0.018	0.129	0.777	2419	tags=24%, list=14%, signal=27%

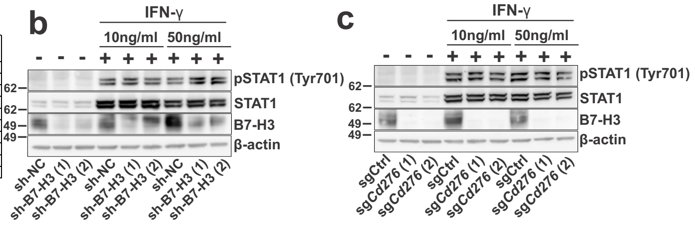

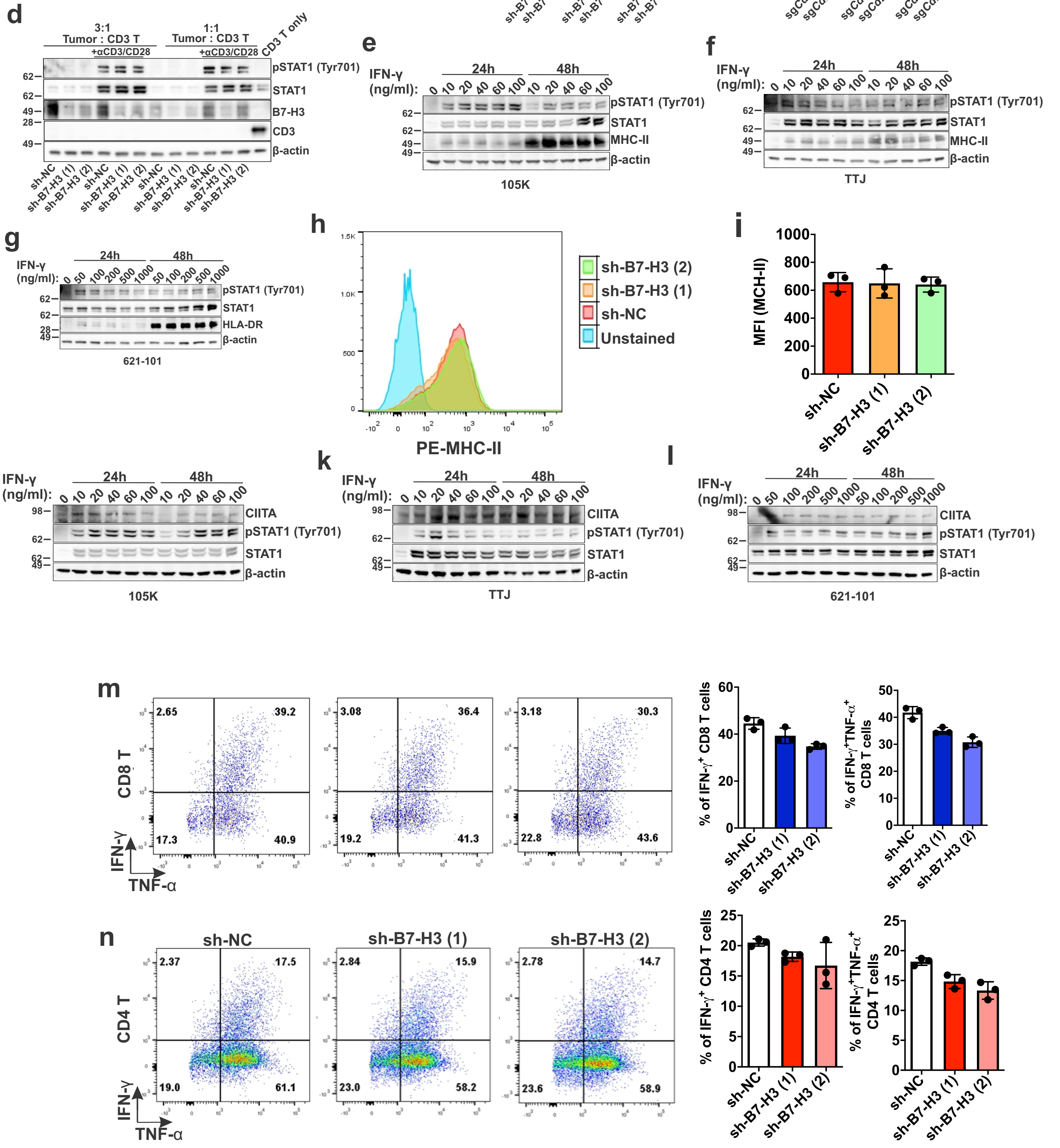

Supplementary Fig. 5. The effects of B7-H3 suppression *in vivo* and *in vitro* relating to IFN-γ.

a, Top 10 activated gene signatures identified by gene set enrichment analysis in B7-H3 knockdown *Tsc2-/-* 105K subcutaneous tumors compared to controls (n = 3). ES = Enrichment Score, NES = Normalized Enrichment Score, NOM = Nominal, FDR = False Discovery Rate.

b, Immunoblot analysis of sh-NC, sh-B7-H3 (1), and sh-B7-H3 (2) *Tsc2-/-* 105K cells treated with IFN-γ for the indicated time points and concentrations (n = 3).

c, Immunoblot analysis of sgCtrl, sg*Cd276* (1), and sg*Cd276* (2) *Tsc2-/-* 105K cells treated with IFN-γ for the indicated time points and concentrations (n = 3).

d, Immunoblot analysis of cells in **(b)** co-cultured with CD3/CD28 activated splenic $CD3^+$ T cells at the indicated tumor: T-cell ratio. CD3 T only was used as a control (n = 3).

e-g, Immunoblot analysis of *Tsc2-/-* 105K cells **(e)**, *Tsc2-/-* TTJ cells **(f)**, and *TSC2-/-* 621-101 cells **(g)** treated with IFN-γ for the indicated concentrations and time durations (n = 3).

h,i, Flow cytometry analysis of MHC-II expression on sh-NC, sh-B7-H3 (1), and sh-B7-H3 (2) *Tsc2-/-* 105K cells. Cells were treated with 10ng/ml of IFN-γ for 48 hr. **(h)**. Mean fluorescent intensity (MFI) of MCH-II measured from each group of cells (n = 3) **(i)**. Means ± SD, one-way ANOVA with Dunnett's multiple comparisons test.

j-l, Immunoblot analysis of *Tsc2-/-* 105K cells **(j)**, *Tsc2-/-* TTJ cells **(k)**, and *TSC2-/-* 621-101 cells **(l)** treated with IFN-γ for the indicated concentrations and time durations (n = 3).

m,n Left panels: Representative flow cytometry plots showing the percentage of IFN-γ/TNF-α-positive splenic $CD8^+$ T cells **(m)** or $CD4^+$ T cells **(n)** incubated with conditioned media isolated from cells in **(b)**. Middle and right panels: Quantification of the left panel. Means ± SD, one-way ANOVA with Dunnett's multiple comparisons test (n = 3).

Source data are provided in the Source data file.

Supplementary Fig. 6

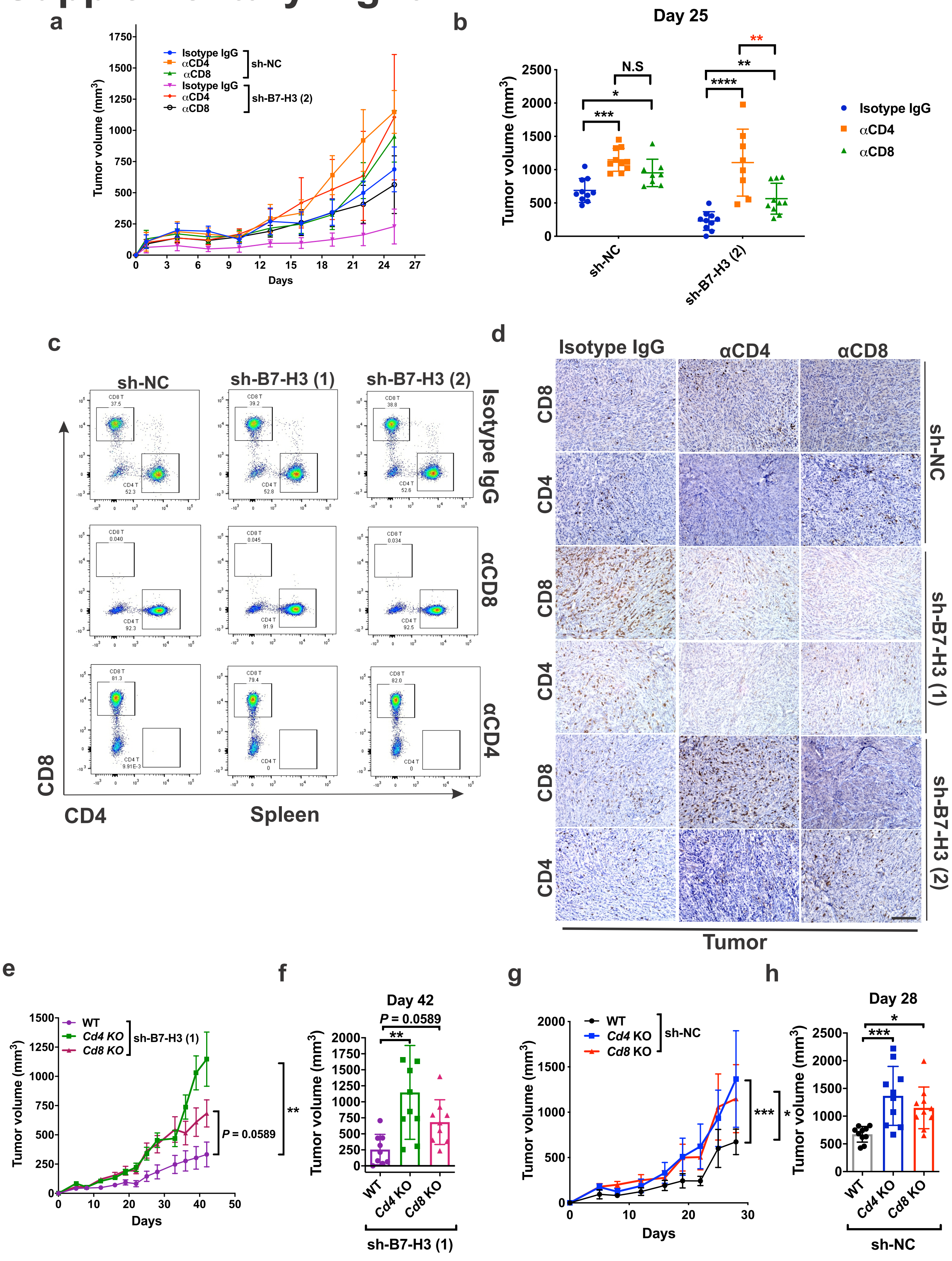

Supplementary Fig. 6. Anti-tumor effect of B7-H3 inhibition depends more on CD4$^+$ T cells compared to CD8$^+$ T cells.

a, Growth of sh-NC or sh-B7-H3 (2) *Tsc2-/-* 105K tumors treated with isotype control, α-CD4 or α-CD8 antibodies (n = 10 mice/group).

b, Tumor volume of (A) at 25-day post-cell injection. n = 10 tumors for sh-NC treated with isotype control, sh-NC treated with α-CD4, and sh-B7-H3 (2) treated with α-CD8, and n = 8 tumors for the remaining groups, means ± SD, two-way ANOVA with Holm-Sidak's multiple comparisons test, * $p < 0.05$, ** $p < 0.01$, *** $p < 0.001$, **** $p < 0.0001$.

c, Representative flow cytometry plots showing that splenic CD8$^+$ and CD4$^+$ T cells were successfully depleted. (n = 5/group).

d, Representative immunohistochemical staining of CD8 and CD4 on tissue sections of sh-NC, sh-B7-H3 (1), or sh-B7-H3 (2) *Tsc2-/-* 105K tumors treated with the indicated antibody. Scale bar = 100 µm. (n = 5/group).

e, Growth of sh-B7-H3 (1) *Tsc2-/-* 105K tumors in WT, *Cd4* KO, or *Cd8* KO mice. n = 9 mice for WT, *Cd8* KO, n = 9 mice for *Cd4* KO, means ± SD, nonparametric Kruskal-Wallis test with Dunn's multiple comparisons test, ** $p < 0.01$.

f, Tumor volume of **e** at 42-day post-cell injection. n = 9 mice for WT, *Cd8* KO, n = 9 mice for *Cd4* KO, means ± SD, nonparametric Kruskal-Wallis test with Dunn's multiple comparisons test, ** $p < 0.01$.

g, Growth of sh-NC *Tsc2-/-* 105K tumors in WT, *Cd4* KO, or *Cd8* KO mice. n = 10 mice/group, means ± SD, nonparametric Kruskal-Wallis test with Dunn's multiple comparisons test, * $p < 0.05$, *** $p < 0.001$.

h, Tumor volume of **g** at 28-day post-cell injection. n = 10 mice/group, means ± SD, nonparametric Kruskal-Wallis test with Dunn's multiple comparisons test, * $p < 0.05$, *** $p < 0.001$.

Source data and exact p values are provided in the Source data file.

Supplementary Fig. 7

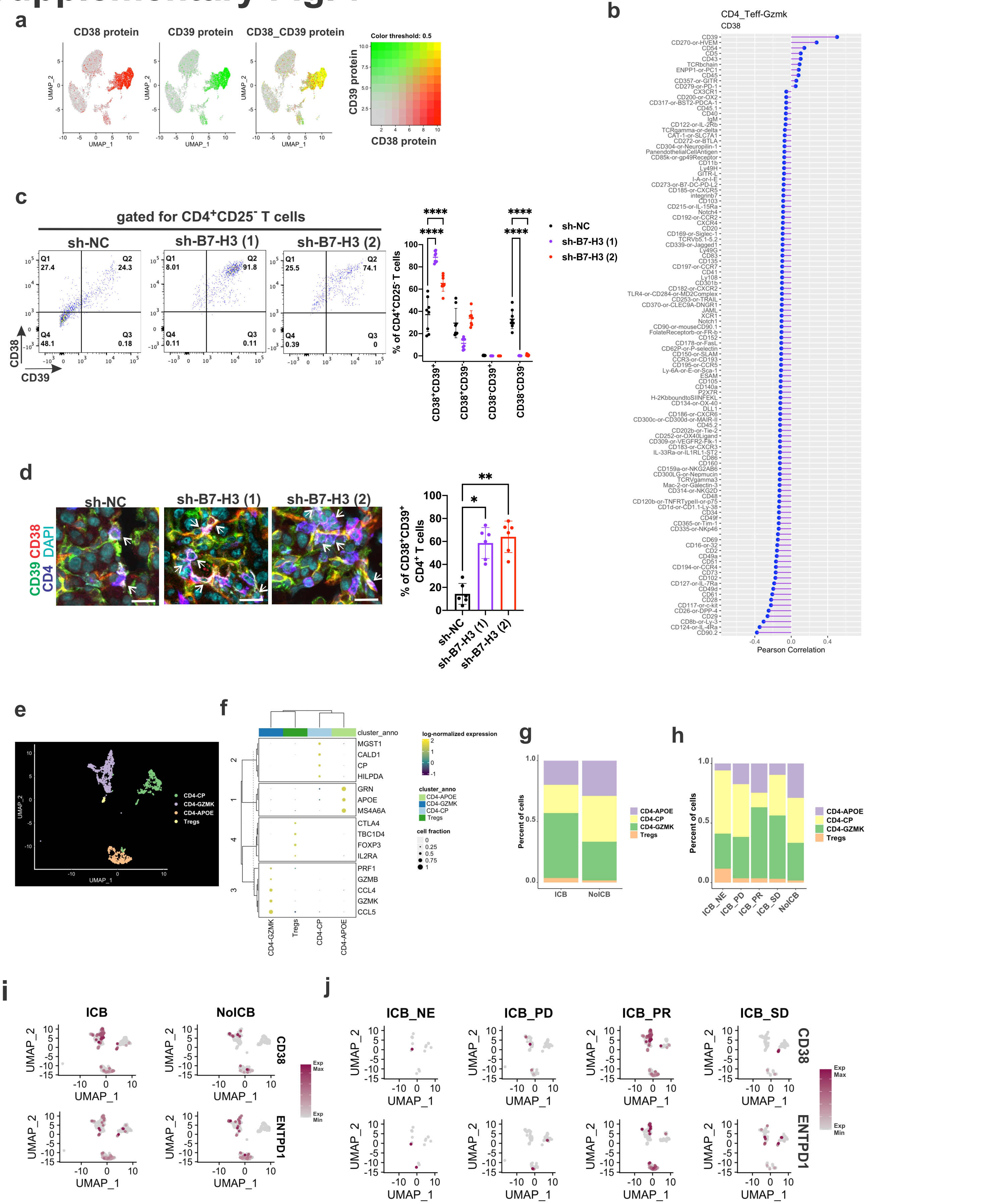

Supplementary Fig. 7. Inhibition of B7-H3 increases the number of intratumoral CD38^{+}CD39^{+}CD4^{+} TILs and analysis of CD4^{+} T cells from advanced metastatic RCC patients.

a, UMAP plots showing co-expression of CD38 and CD39 proteins in CD4_Teff-Gzmk cells.

b, Spearman correlation analysis of CD38 protein expression versus other proteins detected by CITE-seq in CD4_Teff-Gzmk cells.

c, Representative flow cytometry plots (left panel) and quantification of the percentage (right panel) of CD38 and CD39 on CD4^{+}CD25^{-} TILs in sh-NC, sh-B7-H3 (1), and sh-B7-H3 (2) *Tsc2-/-* 105K tumors. n = 8 mice/group, mean ± SD, two-way ANOVA with Holm-Sidak's multiple comparisons test, **** $p < 0.0001$.

d, Representative immunofluoresent images (left panel) and quantification (right panel) of CD38^{+}CD39^{+}CD4^{+} TILs in lungs of WT mice with sh-NC, sh-B7-H3 (1), or sh-B7-H3 (2) *Tsc2-/-* 105K tumors. n = 6 mice/group, mean ± SD, nonparametric Kruskal-Wallis test with Dunn's multiple comparisons test, * $p < 0.05$, ** $p < 0.01$. White arrows showing CD38^{+}CD39^{+}CD4^{+} TILs.

e, UMAP plot of CD4^{+} T cells combined from 8 metastatic RCC patients. 3 from kidneys, 3 from lymph nodes, 2 from visceral metastases.

f, Dotplot showing expression of selected genes from CD4^{+} T cells in **e**.

g,h, Proportions of each CD4^{+} T-cell clusters. ICB = Immune checkpoint blockade, NoICB = No Immune checkpoint blockade. ICB Response: PR = partial response, SD = stable disease, PD = progressive disease, and NE = not evaluable.

i, UMAP plots displaying expression of *CD38* and *ENTPD1* (CD39 gene name) in tumors according to their ICB exposure.

j, UMAP plots displaying expression of *CD38* and *ENTPD1* in tumors according to their ICB response. Source data and exact p values are provided in the Source data file.

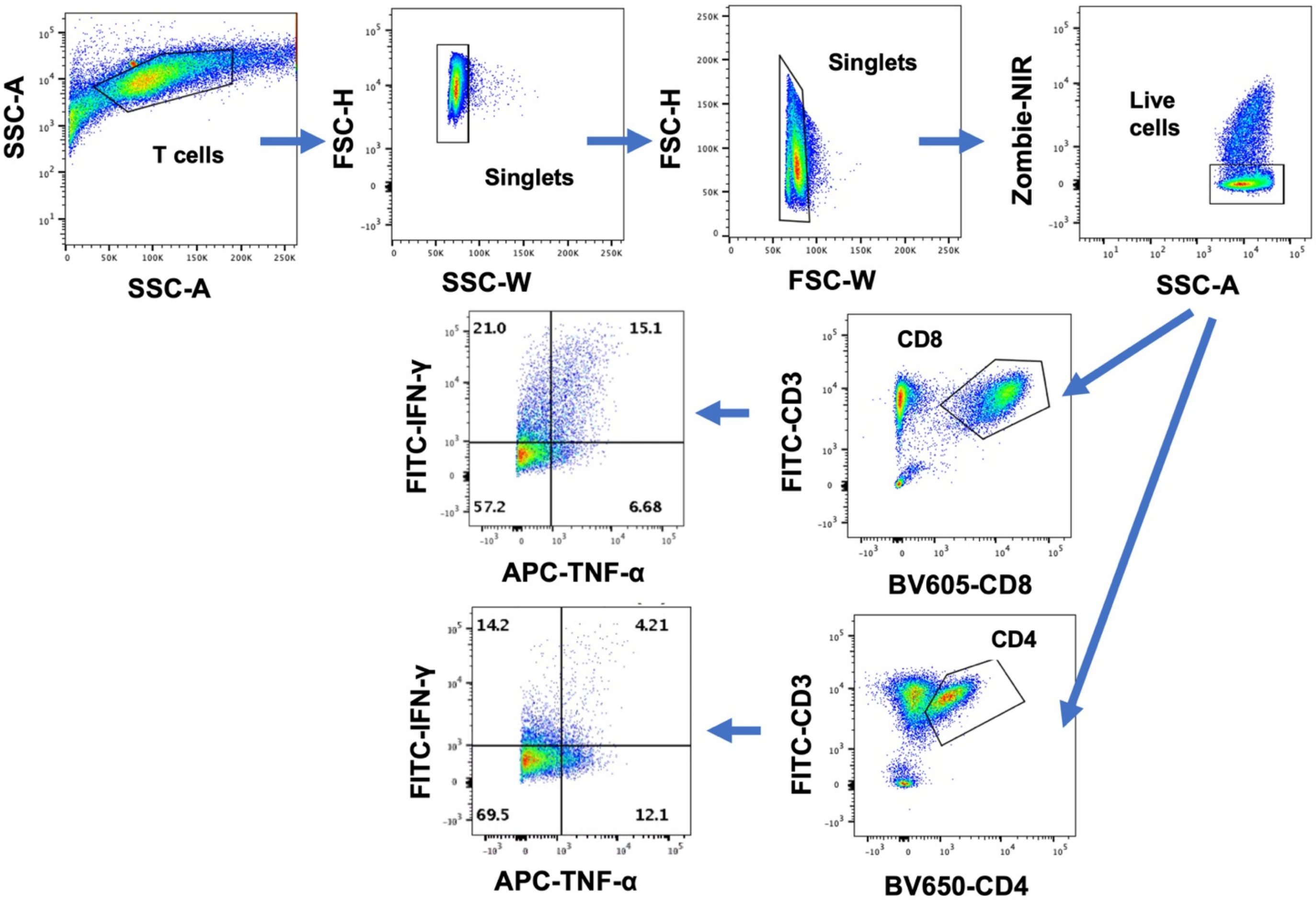

Supplementary Fig 8. Gating strategy for *in vitro* CD3/CD28 activated T-cell IFN-γ and TNF-α analysis.

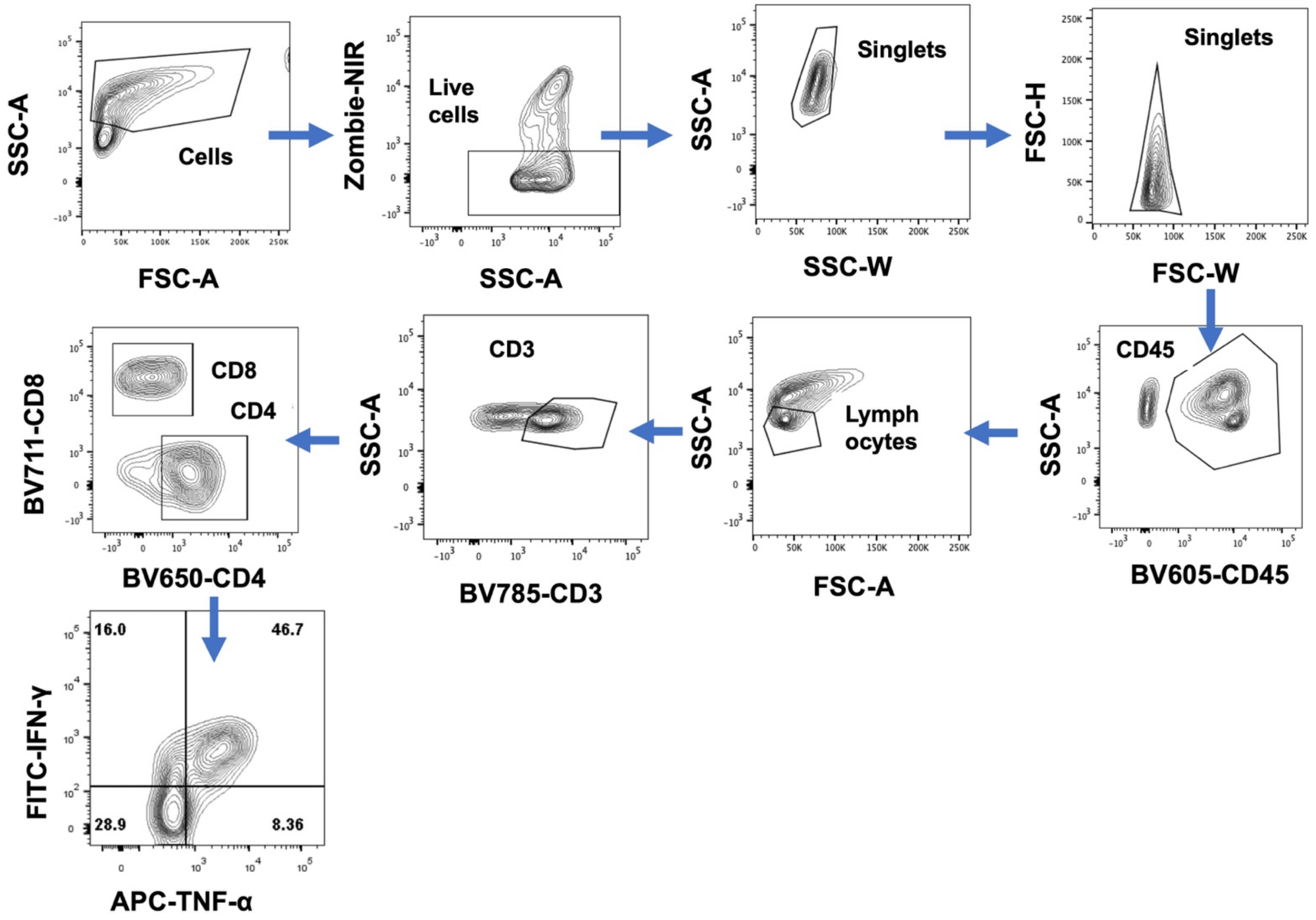

Supplementary Fig 9. Gating strategy for *ex vivo* tumor-infiltrating T-cell IFN-γ and TNF-α analysis.

Supplementary Table 1. List of antibodies used for CyTOF staining

Target	Clone	Supplier	Dilution
CD8α	53-6.7	Lederer Lab CyTOF Core	1:100
CD4	RM4-5	Lederer Lab CyTOF Core	1:100
CD11c	N418	Lederer Lab CyTOF Core	1:100
TCRβ	H57-597	Lederer Lab CyTOF Core	1:100
CD3	145-2C11	Lederer Lab CyTOF Core	1:100
CD19	6D5	Lederer Lab CyTOF Core	1:100
NK1.1	PK136	Lederer Lab CyTOF Core	1:100
CD45	30-F11	Lederer Lab CyTOF Core	1:100
CD11b	M1/70	Lederer Lab CyTOF Core	1:100
Singlec-F	E50-2440	Lederer Lab CyTOF Core	1:100
Ly6G	1A8	Lederer Lab CyTOF Core	1:100
Ly6C	HK1.4	Lederer Lab CyTOF Core	1:100

Supplementary Table 2. List of antibodies used for flow cytometry staining

Target	Clone	Supplier	Dilution
CD45	30-F11	BioLegend	1:100
IFNγ	XMG1.2	BioLegend	1:50
TNF-α	MP6-XT22	BioLegend	1:100
CD4	RM4-5	BioLegend	1:100
CD8	53-6.7	BioLegend	1:100
CD3	17A2	BioLegend	1:100
CD38	90	BioLegend	1:100
CD39	Y23-1185	BD Biosciences	1:100

Supplementary Table 3. List of primers used for ChIP-qPCR

Primer	FW	RV
Cd276 promoter (mouse)	GAGTCCCTTCTTTTCCTTGAGTC	CCCTTCACCTGTGTTTGTATCT

Supplementary Table 4. List of siRNAs used for transfection

siRNA	Cat #	Company
SMARTpools mTOR siRNA	L-065427-00-0005	Dharmacon
SMARTpools Raptor siRNA	L-058754-01-0005	Dharmacon
SMARTpools Rictor siRNA	L-064598-01-0005	Dharmacon
SMARTpools S6K siRNA	L-040893-00-0005	Dharmacon
SMARTpools 4E-BP1 siRNA	L-058681-01-0005	Dharmacon
SMARTpools YY2 siRNA	L-171481-00-0005	Dharmacon
SMARTpools control siRNA	D-001810-10-05	Dharmacon

Supplementary Table 5. List of probes used for qRT-PCR

Probe	Cat #	Company
Cd276 (mouse)	Mm00506020_m1	ThermoFisher Scientific
CD276 (human)	Hs00987207_m1	ThermoFisher Scientific
Yy2 (mouse)	Mm03059489_sH	ThermoFisher Scientific
YY2 (human)	Hs02597954_sH	ThermoFisher Scientific
Yy1 (mouse)	Mm00456392_m1	ThermoFisher Scientific
YY1 (human)	Hs00998747_m1	ThermoFisher Scientific
