# Supplementary material for: mTORC1 upregulates B7-H3/CD276 to inhibit antitumor T cells and drive tumor immune evasion
Source: Nat Commun. 2023 Mar 3;14:1214. doi: 10.1038/s41467-023-36881-7 (PMC9984496; doi:10.1038/s41467-023-36881-7)
Supplement: Supplementary file 3 — Description of Additional Supplementary Files [file 41467_2023_36881_MOESM3_ESM.pdf]

Description of Additional Supplementary Files

Supplementary Data 1: RNAseq B7-H3 KD vs Ctrl ex vivo tumor cells.
Differentially expressed genes in sorted B7-H3 KD tumor cells compared to Ctrl tumor cells. n = 3/group.
