# Supplementary material for: mTORC1 upregulates B7-H3/CD276 to inhibit antitumor T cells and drive tumor immune evasion
Source: Nat Commun. 2023 Mar 3;14:1214. doi: 10.1038/s41467-023-36881-7 (PMC9984496; doi:10.1038/s41467-023-36881-7)
Supplement: Supplementary file 5 — Reporting Summary [file 41467_2023_36881_MOESM5_ESM.pdf]

nature portfolio

Corresponding author(s): Heng-Jia Liu, Elizabeth Henske

Last updated by author(s): 2023/01/10

Reporting Summary

Nature Portfolio wishes to improve the reproducibility of the work that we publish. This form provides structure for consistency and transparency in reporting. For further information on Nature Portfolio policies, see our Editorial Policies and the Editorial Policy Checklist.

Statistics

For all statistical analyses, confirm that the following items are present in the figure legend, table legend, main text, or Methods section.

n/a	Confirmed	
☐	☒	The exact sample size (*n*) for each experimental group/condition, given as a discrete number and unit of measurement
☐	☒	A statement on whether measurements were taken from distinct samples or whether the same sample was measured repeatedly
☐	☒	The statistical test(s) used AND whether they are one- or two-sided<br>*Only common tests should be described solely by name; describe more complex techniques in the Methods section.*
☒	☐	A description of all covariates tested
☐	☒	A description of any assumptions or corrections, such as tests of normality and adjustment for multiple comparisons
☐	☒	A full description of the statistical parameters including central tendency (e.g. means) or other basic estimates (e.g. regression coefficient) AND variation (e.g. standard deviation) or associated estimates of uncertainty (e.g. confidence intervals)
☐	☒	For null hypothesis testing, the test statistic (e.g. *F*, *t*, *r*) with confidence intervals, effect sizes, degrees of freedom and *P* value noted<br>*Give P values as exact values whenever suitable.*
☒	☐	For Bayesian analysis, information on the choice of priors and Markov chain Monte Carlo settings
☒	☐	For hierarchical and complex designs, identification of the appropriate level for tests and full reporting of outcomes
☐	☒	Estimates of effect sizes (e.g. Cohen's *d*, Pearson's *r*), indicating how they were calculated

Our web collection on statistics for biologists contains articles on many of the points above.

Software and code

Policy information about availability of computer code

Data collection	Confocal images were captured using Olympus FV10-FSW software package (v 4.1). IHC images were captured using Keyence BZ-X800 microscope and its integrated viewer. Chemiluminescent images were captured using Syngene G-Box and ThermoFisher iBright 1500 imagers. CD276 reporter dual luciferase activity was analyzed using Biotek Synergy HT running Gen5 v.11.5 software. Gene expression by RT-PCR was analyzed suing AppliedBiosystems Step One Plus real time PCR system (v2.3). RNAseq data was collected using Illumina NextSeq 550. CITE-seq data was collected using NovaSeq 6000.
Data analysis	Image J (v 2.0.0) was used for image analysis. GraphPad Prizm (v6.0 & v.9.3.0) was used for statistical analysis. R package Seurat (v 4.0.2), CellRanger (v 6.0.1), GSVA (v 1.36.3), and DESeq2 (v 1.38.2) were used for data analysis. Genomatix MatInspector software (https://www.genomatix.de) and JASPAR (https://jaspar.genereg.net) were used to identify transcription factors that bind Mouse and human CD276 promoter region.

For manuscripts utilizing custom algorithms or software that are central to the research but not yet described in published literature, software must be made available to editors and reviewers. We strongly encourage code deposition in a community repository (e.g. GitHub). See the Nature Portfolio guidelines for submitting code & software for further information.

Data

Policy information about availability of data

All manuscripts must include a data availability statement. This statement should provide the following information, where applicable:

- Accession codes, unique identifiers, or web links for publicly available datasets
- A description of any restrictions on data availability
- For clinical datasets or third party data, please ensure that the statement adheres to our policy

The raw bulk RNA-seq data generated in this study have been deposited in the GEO database under accession code GSE213626 (https://www.ncbi.nlm.nih.gov/geo/query/acc.cgi?acc=GSE213626). The raw CITE-seq data generated in this study have been deposited under accession code GSE213939 (https://www.ncbi.nlm.nih.gov/geo/query/acc.cgi?acc=GSE213939). The TCGA publicly available data are available in the FireBrowse (http://firebrowse.org). All other data supporting the finding of this study are available within the article and the data generated in this study are provided in the Supplementary Information and Source data file. Source data are provided with this paper.

Human research participants

Policy information about studies involving human research participants and Sex and Gender in Research.

Reporting on sex and gender	N/A
Population characteristics	N/A
Recruitment	N/A
Ethics oversight	N/A

Note that full information on the approval of the study protocol must also be provided in the manuscript.

Field-specific reporting

Please select the one below that is the best fit for your research. If you are not sure, read the appropriate sections before making your selection.

☒ Life sciences ☐ Behavioural & social sciences ☐ Ecological, evolutionary & environmental sciences

For a reference copy of the document with all sections, see nature.com/documents/nr-reporting-summary-flat.pdf

Life sciences study design

All studies must disclose on these points even when the disclosure is negative.

Sample size	For in vitro studies, we used empirical n=3 replicates. Based on large effect size we observed in in vitro studies, we determined that for in vivo study, n=6-10 can achieve a statistic power of 0.8 to detect difference between groups using two-sided t-test at significance of 0.05 at any time points. The exact n for each experiment was described in corresponding figure legends.
Data exclusions	For CITE-seq analysis, cells were filtered from downstream analysis with the criteria of < 200 genes or > 3000 genes detected and > 0.05 fraction of mitochondrial gene.
Replication	All experiments in this study were repeated with at least in 3 biological replicates. All replicates showed similar results.
Randomization	All mice and cell culture experiments were randomly assigned to different groups.
Blinding	All data acquisition and analysis in this study were performed in a blinded way.

Reporting for specific materials, systems and methods

We require information from authors about some types of materials, experimental systems and methods used in many studies. Here, indicate whether each material, system or method listed is relevant to your study. If you are not sure if a list item applies to your research, read the appropriate section before selecting a response.

Materials & experimental systems

n/a	Involved in the study
☐	☒ Antibodies
☐	☒ Eukaryotic cell lines
☒	☐ Palaeontology and archaeology
☐	☒ Animals and other organisms
☒	☐ Clinical data
☒	☐ Dual use research of concern

Methods

n/a	Involved in the study
☒	☐ ChIP-seq
☐	☒ Flow cytometry
☒	☐ MRI-based neuroimaging

Antibodies

Antibodies used

For in vivo treatment:
InVivomAb anti-mouse B7-H3 (clone MJ18) (BioXCell, BE0124, Rat, 300ug)
InVivoMAb Rat IgG1 isotype control (HRPN) (BioXCell, BE0088, Rat, 300ug)
InVivomAb anti-mouse CD4 (clone GK1.5) (BioXCell, BE0003, Rat, 200ug)
InVivomAb anti-mouse CD8 (clone YTS 169.4) (BioXCell, BE0117, Rat, 200ug)
InVivomAb rat IgG2b isotype (LTF-2) (BioXCell, BE0090, Rat, 200ug)
InVivomAb rat IgG1 isotype (HRPN) (BioXCell, BE0088, Rat, 200ug)

For WB/IHC/IF:
Anti-Phospho-p70 S6 Kinase T389 (Cell Signaling Technology, 9234, Rb, 1:1000)
Anti-p70 S6 Kinase (Cell Signaling Technology, 2708, Rb, 1:1000)
Anti-TSC2 (Cell Signaling Technology, 4308, Rb, 1:1000)
Anti-Phospho-S6 S235/236 (Cell Signaling Technology, 2211, Rb, 1:1000)
Anti-S6 (Cell Signaling Technology, 2217, Rb, 1:1000)
Anti-mTOR (Cell Signaling Technology, 2983, Rb, 1:1000)
Anti-Raptor (Cell Signaling Technology, 2280, Rb, 1:1000)
Anti-Rictor (Cell Signaling Technology, 2114, Rb, 1:1000)
Anti-Phospho-Akt S473 (Cell Signaling Technology, 4060, Rb, 1:1000)
Anti-Akt (Cell Signaling Technology, 4685, Rb, 1:1000)
Anti-GAPDH (Cell Signaling Technology, 2118, Rb, 1:1000)
Anti-CREB (Cell Signaling Technology, 4820, Rb, 1:1000)
Anti-STAT1 (Cell Signaling Technology, 14994, Rb, 1:1000)
Anti-p-STAT1 (Tyr 701) (Cell Signaling Technology, 9167, Rb, 1:1000)
Anti-B7-H3 (Human) (Cell Signaling Technology, 14058, Rb, 1:1000)
Anti-YY1 (Cell Signaling Technology, 46395, Rb, 1:1000)
Anti-HA-tag (Cell Signaling Technology, 3724, Rb, 1:1000)
Anti-Phospho-substrate (RXXS*/T*) (110B7E) (Cell Signaling Technology, 9614, Rb, 1:1000)
Anti-E-cadherin (Cell Signaling Technology, 14472, Rb, 1:400)
Anti-F4/80 (Cell Signaling Technology, 70076, Rb, 1:500)
Anti-MHC-II (LSBio, LS-C204829, Rat, 1:100)
Anti-β-actin (Sigma, A1978, Mouse, 1:1000)
Anti-B7-H3 Mouse (R&D Systems, AF1397, Goat, 1:1000)
Anti-YY2 (A-5) Mouse (Santa Cruz, sc-377008, Mouse, 1:1000)
Anti-YY2 (C-10) Human (Santa Cruz, sc-374455, Mouse, 1:1000)
Anti-CIITA (Santa Cruz, sc-13556, Mouse, 1:1000)
Anti-CD31 (Abcam, ab182981, Rb, 1:500)
Anti-GFP (Abcam, ab6556, Rb, 1:1000)
Anti-CD4 (eBioscience, 14-9766-82, Rat, 1:1000)
Anti-CD8 (eBioscience, 14-0808-82, Rat, 1:1000)
Anti-rabbit IgG (Vector Laboratories, MP7451)
Anti-mouse IgG (Vector Laboratories, MP7452)
Anti-CD38 Alexa Fluor 594 (90) Mouse (BioLegend,102725, Rat, 1:100)
Anti-CD39 (EPR22507-48) Mouse (Abcam, Ab227840, Rb, 1:250)
Anti-Myc Tag (Cell Signaling Technology, 2276, Mouse, 1:1000)
Anti-DYKDDDDK Tag (Cell Signaling Technology, 8146, Mouse, 1:1000)
goat anti-rabbit IgG H&L(Alexa Fluor 488) (ThermoFisher, A-11008, 1:500)
goat anti-rabbit IgG H&L(Alexa Fluor 594) (ThermoFisher, A-11012, 1:500)
goat anti-rabbit IgG H&L(Alexa Fluor 647) (ThermoFisher, A-21245, 1:500)

For Flow Cytometry/CyTOF:
Anti-Ly6G PerCP (1A8) Mouse (BioLegend, 127654, Rat, 1:100)
Anti-CD11c BV510 (N418) Mouse (BioLegend, 117338, Rat 1:100)
Anti-Ly6C BV605 (HK1.4) Mouse (BioLegend, 128036, Rat) 1:100
Anti-CD45 BV711 (30-F11) Mouse (BioLegend, 103147, Rat 1:100)

Anti-CD45 BV605 (30-F11) Mouse (BioLegend, 103140, Rat 1:100)
Anti-CD11b BV785 (M1/70) Mouse (BioLegend, 101243, Rat 1:100)
Anti-IFN-γ FITC (XMG1.2) Mouse (BioLegend, 505806, Rat ,1:50)
Anti-TNF-α APC (MP6-XT22) Mouse (BioLegend, 506308, Rat, 1:100)
Anti-CD4 BV650 (RM4-5) Mouse (BioLegend, 100546, Rat, 1:100)
Anti-CD8 BV711 (53-6.7) Mouse (BioLegend, 100748, Rat, 1:100)
Anti-CD3 BV785 (17A2) Mouse (BioLegend, 100232, Rat, 1:100)
Anti-CD3 FITC (17A2) Mouse (BioLegend, 100204, Rat, 1:100)
Anti-CD4 Alexa Fluor 647 (GK1.5) Mouse (BioLegend, 100424, Rat, 1:100)
Anti-CD38 BV421 (90) Mouse (BioLegend, 102732, Rat, 1:100)
Anti-Rat IgG2b, k Isotype (BioLegend, 400601, Rat, 1:100)
Anti-I-A/I-E (M5/114.15.2) (BioLegend, 107601, Rat, 1:100)
Anti-CD39 PE (Y23-1185) Mouse (BD Biosciences, 567104, Rat, 1:100)
Anti-CD8α 142Nd (53-6.7) Mouse (Lederer Lab CyTOF Core, 1:100)
Anti-CD4 145Nd (RM4-5) Mouse (Lederer Lab CyTOF Core, 1:100)
Anti-CD11c 146Nd (N418) Mouse (Lederer Lab CyTOF Core, 1:100)
Anti-TCRβ 150Nd (H57-597) Mouse (Lederer Lab CyTOF Core, 1:100)
Anti-CD3 152Sm (145-2C11) Mouse (Lederer Lab CyTOF Core, 1:100)
Anti-CD19 156Gd (6D5) Mouse (Lederer Lab CyTOF Core, 1:100)
Anti-NK1.1 163 Dy (PK136) Mouse (Lederer Lab CyTOF Core, 1:100)
Anti-CD45 165Ho (30-F11) Mouse (Lederer Lab CyTOF Core, 1:100)
Anti-Anti-CD11b 169Tm (M1/70) Mouse (Lederer Lab CyTOF Core, 1:100)
Anti-Singlec-F 170Er (E50-2440) Mouse (Lederer Lab CyTOF Core, 1:100)
Anti-Ly6G 158Gd (1A8) Mouse (Lederer Lab CyTOF Core, 1:100)
Anti-Ly6C 151Eu (HK1.4) Mouse (Lederer Lab CyTOF Core, 1:100)

For CITE-seq:
TotalSeq-A lyophilized antibodies Mouse (BioLegend, 99833, 1 vial per 5 x 105 cell)
TrueStain FcX Antibody (BioLegend, 101320, Rat, 1:100)

Validation

All antibodies used in this study are commercially available and all are validated by the vendors for the specific assays and species used; the validation data is available on the vendors website.
1. InVivomAb anti-mouse B7-H3 (clone MJ18) (BioXCell, BE0124, Rat)
https://bioxcell.com/invivomab-anti-mouse-cd276-b7-h3-be0124
The manufacturer has validated this antibody for in vivo use in species mouse.
2. InVivoMAb Rat IgG1 isotype control (HRPN) (BioXCell, BE0088, Rat)
https://bioxcell.com/invivomab-rat-igg1-isotype-control-anti-horseradish-peroxidase-be0088
The manufacturer has validated this antibody for in vivo use in species mouse.
3. InVivomAb anti-mouse CD4 (clone GK1.5) (BioXCell, BE0003, Rat)
https://bioxcell.com/invivomab-anti-mouse-cd4-be0003-1
The manufacturer has validated this antibody for in vivo use in species mouse.
4. InVivomAb anti-mouse CD8 (clone YTS 169.4) (BioXCell, BE0117, Rat)
https://bioxcell.com/invivomab-anti-mouse-cd8a-be0117
The manufacturer has validated this antibody for in vivo use in species mouse.
5. InVivomAb rat IgG2b isotype (LTF-2) (BioXCell, BE0090, Rat)
https://bioxcell.com/invivomab-rat-igg2b-isotype-control-anti-keyhole-limpet-hemocyanin-be0090
The manufacturer has validated this antibody for in vivo use in species mouse.
6. InVivomAb rat IgG1 isotype (HRPN) (BioXCell, BE0088, Rat)
https://bioxcell.com/invivomab-rat-igg1-isotype-control-anti-horseradish-peroxidase-be0088
The manufacturer has validated this antibody for in vivo use in species mouse.
7. Anti-Phospho-p70 S6 Kinase T389 (Cell Signaling Technology, 9234, Rb)
https://www.cellsignal.com/product/productDetail.jsp?productId=9234
The manufacturer has validated this antibody for WB in the species human and mouse.
8. Anti-p70 S6 Kinase (Cell Signaling Technology, 2708, Rb)
https://www.cellsignal.com/product/productDetail.jsp?productId=2708
The manufacturer has validated this antibody for WB in the species human and mouse.
9. Anti-TSC2 (Cell Signaling Technology, 4308, Rb)
https://www.cellsignal.com/product/productDetail.jsp?productId=4308
The manufacturer has validated this antibody for WB in the species human and mouse.
10. Anti-Phospho-S6 S235/236 (Cell Signaling Technology, 2211, Rb)
https://www.cellsignal.com/product/productDetail.jsp?productId=2211

The manufacturer has validated this antibody for WB in the species human and mouse.
11. Anti-S6 (Cell Signaling Technology, 2217, Rb)
https://www.cellsignal.com/product/productDetail.jsp?productId=2217
The manufacturer has validated this antibody for WB in the species human and mouse.
12. Anti-mTOR (Cell Signaling Technology, 2983, Rb)
https://www.cellsignal.com/product/productDetail.jsp?productId=2983
The manufacturer has validated this antibody for WB in the species human and mouse.
13. Anti-Raptor (Cell Signaling Technology, 2280, Rb)
https://www.cellsignal.com/product/productDetail.jsp?productId=2280
The manufacturer has validated this antibody for WB in the species human and mouse.
14. Anti-Rictor (Cell Signaling Technology, 2114, Rb)
https://www.cellsignal.com/product/productDetail.jsp?productId=2114
The manufacturer has validated this antibody for WB in the species human and mouse.
15. Anti-Phospho-Akt S473 (Cell Signaling Technology, 4060, Rb)
https://www.cellsignal.com/product/productDetail.jsp?productId=4060
The manufacturer has validated this antibody for WB in the species human and mouse.
16. Anti-Akt (Cell Signaling Technology, 4685, Rb)
https://www.cellsignal.com/product/productDetail.jsp?productId=4685
The manufacturer has validated this antibody for WB in the species human and mouse.
17. Anti-GAPDH (Cell Signaling Technology, 2118, Rb)
https://www.cellsignal.com/product/productDetail.jsp?productId=2118
The manufacturer has validated this antibody for WB in the species human and mouse.
18. Anti-CREB (Cell Signaling Technology, 4820, Rb)
https://www.cellsignal.com/product/productDetail.jsp?productId=4820
The manufacturer has validated this antibody for WB in the species human and mouse.
19. Anti-STAT1 (Cell Signaling Technology, 14994, Rb)
https://www.cellsignal.com/product/productDetail.jsp?productId=14994
The manufacturer has validated this antibody for WB/IHC in the species human and mouse.
20. Anti-p-STAT1 (Tyr 701) (Cell Signaling Technology, 9167, Rb)
https://www.cellsignal.com/product/productDetail.jsp?productId=9167
The manufacturer has validated this antibody for WB in the species human and mouse.
21. Anti-B7-H3 (Human) (Cell Signaling Technology, 14058, Rb)
https://www.cellsignal.com/product/productDetail.jsp?productId=14058
The manufacturer has validated this antibody for WB/IHC in the species human.
22. Anti-YY1 (Cell Signaling Technology, 46395, Rb)
https://www.cellsignal.com/product/productDetail.jsp?productId=46395
The manufacturer has validated this antibody for WB in the species human.
23. Anti-HA-tag (Cell Signaling Technology, 3724, Rb)
https://www.cellsignal.com/product/productDetail.jsp?productId=3724
The manufacturer has validated this antibody for WB in the species human and mouse.
24. Anti-Phospho-substrate (RXXS*/T*) (110B7E) (Cell Signaling Technology, 9614, Rb)
https://www.cellsignal.com/product/productDetail.jsp?productId=9614
The manufacturer has validated this antibody for WB in the species human and mouse.
25. Anti-E-cadherin (Cell Signaling Technology, 14472, Rb)
https://www.cellsignal.com/products/primary-antibodies/e-cadherin-4a2-mouse-mab/14472?_=1673373457991&Ntt=e-cad&tahead=true
The manufacturer has validated this antibody for WB/IHC in the species human and mouse.
26. Anti-F4/80 (Cell Signaling Technology, 70076, Rb)
https://www.cellsignal.com/product/productDetail.jsp?productId=70076
The manufacturer has validated this antibody for IHC in the species mouse.
27. Anti-MHC-II (LSBio, LS-C204829, Rat)
https://www.lsbio.com/antibodies/mhc-class-ii-i-a-antibody-i-e-antibody-clone-m5-114-azide-free-flow-ihc-ip-wb-western-ls-c204829/213397
The manufacturer has validated this antibody for WB/IHC in the species mouse.
28. Anti-β-actin (Sigma, A1978, Mouse)
https://www.sigmaaldrich.com/US/en/product/sigma/a1978?gclid=Cj0KCQiAtvSdBhD0ARIsAPf8oNm0z020fBdVs6QUJpkkfnGVpQk00P4YCYtCze0LI2RtMM8FH4gSED8aAtZ2EALw_wcB&gclsrc=aw.ds
The manufacturer has validated this antibody for WB in the species human and mouse.
29. Anti-B7-H3 Mouse (R&D Systems, AF1397, Goat)
https://www.rndsystems.com/products/mouse-b7-h3-antibody_af1397
The manufacturer has validated this antibody for WB in the species mouse.
30. Anti-YY2 (A-5) Mouse (Santa Cruz, sc-377008, Mouse)
https://www.scbt.com/p/yy2-antibody-a-5
The manufacturer has validated this antibody for WB in the species mouse.
31. Anti-YY2 (C-10) Human (Santa Cruz, sc-374455, Mouse)
https://www.scbt.com/p/yy2-antibody-c-10
The manufacturer has validated this antibody for WB in the species human.
32. Anti-CIITA (Santa Cruz, sc-13556, Mouse)

https://www.scbt.com/p/ciita-antibody-7-1h
The manufacturer has validated this antibody for WB in the species human and mouse.
33. Anti-CD31 (Abcam, ab182981, Rb)
https://www.abcam.com/cd31-antibody-epr17259-ab182981.html
The manufacturer has validated this antibody for IHC in the species human and mouse.
34. Anti-GFP (Abcam, ab6556, Rb)
https://www.abcam.com/gfp-antibody-ab6556.html
The manufacturer has validated this antibody for WB in the species human and mouse.
35. Anti-CD4 (eBioscience, 14-9766-82, Rat)
https://www.thermofisher.com/antibody/product/CD4-Antibody-clone-4SM95-Monoclonal/14-9766-82
The manufacturer has validated this antibody for IHC in the species mouse.
36. Anti-CD8 (eBioscience, 14-0808-82, Rat)
https://www.thermofisher.com/antibody/product/CD8a-Antibody-clone-4SM15-Monoclonal/14-0808-82
The manufacturer has validated this antibody for IHC in the species mouse.
37. Anti-rabbit IgG (Vector Laboratories, MP7451)
https://vectorlabs.com/products/enzyme-polymer/immpress-hrp-goat-anti-rabbit-igg-kit
The manufacturer has validated this antibody for IHC in the species human and mouse.
38. Anti-mouse IgG (Vector Laboratories, MP7452)
https://vectorlabs.com/products/enzyme-polymer/immpress-hrp-goat-anti-mouse-igg-kit
The manufacturer has validated this antibody for IHC in the species human and mouse.
39. Anti-CD38 Alexa Fluor 594 (90) Mouse (BioLegend,102725, Rat)
https://www.biolegend.com/fr-fr/products/alexa-fluor-594-anti-mouse-cd38-antibody-12447?GroupID=BLG247
The manufacturer has validated this antibody for IHC in the species mouse.
40. Anti-CD39 (EPR22507-48) Mouse (Abcam, Ab227840, Rb)
https://www.abcam.com/cd39-antibody-epr22507-48-ab227840.html
The manufacturer has validated this antibody for IHC in the species mouse.
41. Anti-Myc Tag (Cell Signaling Technology, 2276, Mouse)
https://www.cellsignal.com/products/primary-antibodies/myc-tag-9b11-mouse-mab/2276
The manufacturer has validated this antibody for WB in the species human and mouse.
42. Anti-DYKDDDDK Tag (Cell Signaling Technology, 8146, Mouse)
https://www.cellsignal.com/products/primary-antibodies/dykddddk-tag-9a3-mouse-mab-binds-to-same-epitope-as-sigma-s-anti-flag-m2-antibody/8146
The manufacturer has validated this antibody for WB in the species human and mouse.
43. goat anti-rabbit IgG H&L(Alexa Fluor 488) (ThermoFisher, A-11008)
https://www.thermofisher.com/antibody/product/Goat-anti-Rabbit-IgG-H-L-Cross-Adsorbed-Secondary-Antibody-Polyclonal/A-11008
The manufacturer has validated this antibody for IF in the species human and mouse.
44. goat anti-rabbit IgG H&L(Alexa Fluor 594) (ThermoFisher, A-11012)
https://www.thermofisher.com/antibody/product/Goat-anti-Rabbit-IgG-H-L-Cross-Adsorbed-Secondary-Antibody-Polyclonal/A-11012
The manufacturer has validated this antibody for IF in the species human and mouse.
45. goat anti-rabbit IgG H&L(Alexa Fluor 647) (ThermoFisher, A-21245)
https://www.thermofisher.com/antibody/product/Goat-anti-Rabbit-IgG-H-L-Highly-Cross-Adsorbed-Secondary-Antibody-Polyclonal/A-21245
The manufacturer has validated this antibody for IF in the species human and mouse.
46. Anti-Ly6G PerCP (1A8) Mouse (BioLegend, 127654, Rat)
https://www.biolegend.com/nl-nl/products/percp-anti-mouse-ly-6g-antibody-13351
The manufacturer has validated this antibody for FC in the species mouse.
47. Anti-CD11c BV510 (N418) Mouse (BioLegend, 117338, Rat)
https://www.biolegend.com/nl-nl/products/brilliant-violet-510-anti-mouse-cd11c-antibody-8491
The manufacturer has validated this antibody for FC in the species mouse.
48. Anti-Ly6C BV605 (HK1.4) Mouse (BioLegend, 128036, Rat)
https://www.biolegend.com/nl-nl/products/brilliant-violet-605-anti-mouse-ly-6c-antibody-8727
The manufacturer has validated this antibody for FC in the species mouse.
49. Anti-CD45 BV711 (30-F11) Mouse (BioLegend, 103147, Rat)
https://www.biolegend.com/nl-nl/products/brilliant-violet-711-anti-mouse-cd45-antibody-10439
The manufacturer has validated this antibody for FC in the species mouse.
50. Anti-CD45 BV605 (30-F11) Mouse (BioLegend, 103140, Rat)
https://www.biolegend.com/nl-nl/products/brilliant-violet-605-anti-mouse-cd45-antibody-8721
The manufacturer has validated this antibody for FC in the species mouse.
51. Anti-CD11b BV785 (M1/70) Mouse (BioLegend, 101243, Rat)
https://www.biolegend.com/nl-nl/products/brilliant-violet-785-anti-mouse-human-cd11b-antibody-7958
The manufacturer has validated this antibody for FC in the species mouse.
52. Anti-IFN-γ FITC (XMG1.2) Mouse (BioLegend, 505806, Rat)
https://www.biolegend.com/nl-nl/products/fitc-anti-mouse-ifn-gamma-antibody-995
The manufacturer has validated this antibody for FC in the species mouse.
53. Anti-TNF-α APC (MP6-XT22) Mouse (BioLegend, 506308, Rat)
https://www.biolegend.com/nl-nl/products/apc-anti-mouse-tnf-alpha-antibody-975
The manufacturer has validated this antibody for FC in the species mouse.

54. Anti-CD4 BV650 (RM4-5) Mouse (BioLegend, 100546, Rat)
https://www.biolegend.com/nl-nl/products/brilliant-violet-650-anti-mouse-cd4-antibody-7634
The manufacturer has validated this antibody for FC in the species mouse.
55. Anti-CD8 BV711 (53-6.7) Mouse (BioLegend, 100748, Rat)
https://www.biolegend.com/nl-nl/products/brilliant-violet-711-anti-mouse-cd8a-antibody-7926
The manufacturer has validated this antibody for FC in the species mouse.
56. Anti-CD3 BV785 (17A2) Mouse (BioLegend, 100232, Rat)
https://www.biolegend.com/nl-nl/products/brilliant-violet-785-anti-mouse-cd3-antibody-7953
The manufacturer has validated this antibody for FC in the species mouse.
57. Anti-CD3 FITC (17A2) Mouse (BioLegend, 100204, Rat)
https://www.biolegend.com/nl-nl/products/fitc-anti-mouse-cd3-antibody-45
The manufacturer has validated this antibody for FC in the species mouse.
58. Anti-CD4 Alexa Fluor 647 (GK1.5) Mouse (BioLegend, 100424, Rat)
https://www.biolegend.com/nl-nl/products/alexa-fluor-647-anti-mouse-cd4-antibody-2694
The manufacturer has validated this antibody for FC in the species mouse.
59. Anti-CD38 BV421 (90) Mouse (BioLegend, 102732, Rat)
https://www.biolegend.com/nl-nl/products/brilliant-violet-421-anti-mouse-cd38-antibody-15883
The manufacturer has validated this antibody for FC in the species mouse.
60. Anti-Rat IgG2b, k Isotype (BioLegend, 400601, Rat)
https://www.biolegend.com/nl-nl/products/purified-rat-igg2b-kappa-isotype-ctrl-1858
The manufacturer has validated this antibody for FC in the species mouse.
61. Anti-I-A/I-E (M5/114.15.2) (BioLegend, 107601, Rat)
https://www.biolegend.com/nl-nl/products/purified-anti-mouse-i-a-i-e-antibody-368
The manufacturer has validated this antibody for FC in the species mouse.
62. Anti-CD39 PE (Y23-1185) Mouse (BD Biosciences, 567104, Rat)
https://www.bdbiosciences.com/en-eu/products/reagents/flow-cytometry-reagents/research-reagents/single-color-antibodies-ruo/pe-rat-anti-mouse-cd39.567104
The manufacturer has validated this antibody for FC in the species mouse.
63. Anti-CD8α 142Nd (53-6.7) Mouse (Lederer Lab CyTOF Core)
https://ledererlab.bwh.harvard.edu/cytof-core/
The manufacturer has validated this antibody for CyTOF in the species mouse.
64. Anti-CD4 145Nd (RM4-5) Mouse (Lederer Lab CyTOF Core)
https://ledererlab.bwh.harvard.edu/cytof-core/
The manufacturer has validated this antibody for CyTOF in the species mouse.
65. Anti-CD11c 146Nd (N418) Mouse (Lederer Lab CyTOF Core)
https://ledererlab.bwh.harvard.edu/cytof-core/
The manufacturer has validated this antibody for CyTOF in the species mouse.
66. Anti-TCRβ 150Nd (H57-597) Mouse (Lederer Lab CyTOF Core)
https://ledererlab.bwh.harvard.edu/cytof-core/
The manufacturer has validated this antibody for CyTOF in the species mouse.
67. Anti-CD3 152Sm (145-2C11) Mouse (Lederer Lab CyTOF Core)
https://ledererlab.bwh.harvard.edu/cytof-core/
The manufacturer has validated this antibody for CyTOF in the species mouse.
68. Anti-CD19 156Gd (6D5) Mouse (Lederer Lab CyTOF Core)
https://ledererlab.bwh.harvard.edu/cytof-core/
The manufacturer has validated this antibody for CyTOF in the species mouse.
69. Anti-NK1.1 163 Dy (PK136) Mouse (Lederer Lab CyTOF Core)
https://ledererlab.bwh.harvard.edu/cytof-core/
The manufacturer has validated this antibody for CyTOF in the species mouse.
70. Anti-CD45 165Ho (30-F11) Mouse (Lederer Lab CyTOF Core)
https://ledererlab.bwh.harvard.edu/cytof-core/
The manufacturer has validated this antibody for CyTOF in the species mouse.
71. Anti-Anti-CD11b 169Tm (M1/70) Mouse (Lederer Lab CyTOF Core)
https://ledererlab.bwh.harvard.edu/cytof-core/
The manufacturer has validated this antibody for CyTOF in the species mouse.
72. Anti-Singlec-F 170Er (E50-2440) Mouse (Lederer Lab CyTOF Core)
https://ledererlab.bwh.harvard.edu/cytof-core/
The manufacturer has validated this antibody for CyTOF in the species mouse.
73. Anti-Ly6G 158Gd (1A8) Mouse (Lederer Lab CyTOF Core)
https://ledererlab.bwh.harvard.edu/cytof-core/
The manufacturer has validated this antibody for CyTOF in the species mouse.
74. Anti-Ly6C 151Eu (HK1.4) Mouse (Lederer Lab CyTOF Core)
https://ledererlab.bwh.harvard.edu/cytof-core/
The manufacturer has validated this antibody for CyTOF in the species mouse.
75. TotalSeq-A lyophilized antibodies Mouse (BioLegend, 99833)
The manufacturer has validated this antibody for CITE-seq in the species mouse.
76. TrueStain FcX Antibody (BioLegend, 101320, Rat)
https://www.biolegend.com/en-us/products/trustain-fcx-anti-mouse-cd16-32-antibody-5683
The manufacturer has validated this antibody for Fc blocking in the species mouse.

Eukaryotic cell lines

Policy information about cell lines and Sex and Gender in Research

Cell line source(s)	Tsc2−/− 105K renal tumor cells stably expressing empty vector or TSC2, Tsc2 WT and Tsc2 KO MEFs, 621-101 cells stably expressing empty vector or TSC2 were generated in-house. Tsc2−/− TTJ renal tumor cells was a gift from Vera Krymskaya. A549, T47D and PC3 cells were purchased from ATCC. Raptor and Rictor inducible MEFs were the kind gifts from Michael Hall.
Authentication	TSC2-null and TSC2 add-back cell lines are routinely authenticated using western blotting and qPCR to confirm TSC2 protein loss and expression before conducting all experiments. All other cell lines were authenticated by the vendor.
Mycoplasma contamination	Cells were tested for mycoplasma every month and were confirmed negative.
Commonly misidentified lines (See ICLAC register)	No commonly misidentified cell lines were used in the study.

Animals and other research organisms

Policy information about studies involving animals; ARRIVE guidelines recommended for reporting animal research, and Sex and Gender in Research

Laboratory animals	6-8 week-old C57BL/6J, Ifngtm1Ts/J (Ifng KO), Rag1tm1Mom/J (Rag1 KO), Cd4tm1Mak/J (CD4 KO), Cd8atm1Mak/J (CD8 KO) and 7-month-old AJ Tsc2+/- mice were used.
Wild animals	No wild-animals were used in this study.
Reporting on sex	Sex and gender were not considered in study design.
Field-collected samples	No Field-collected samples were used in this study.
Ethics oversight	All animal procedures were approved by the Brigham and Women's Hospital Institutional Animal Care and Use Committee.

Note that full information on the approval of the study protocol must also be provided in the manuscript.

Flow Cytometry

Plots

Confirm that:

- [x] The axis labels state the marker and fluorochrome used (e.g. CD4-FITC).
- [x] The axis scales are clearly visible. Include numbers along axes only for bottom left plot of group (a 'group' is an analysis of identical markers).
- [x] All plots are contour plots with outliers or pseudocolor plots.
- [x] A numerical value for number of cells or percentage (with statistics) is provided.

Methodology

Sample preparation	S.c. solid tumors were disrupted mechanically with a surgical scalpel and then enzymatically digested in HBSS (Invitrogen) containing 400 U/ml collagenase IV and 20U/ml DNase I, with constant rocking at 37oC for 45 minutes. The resulting cell suspension was passed through a 70-μm cell strainer and washed once with 1× PBS containing 0.4% FBS and 2.5 mM EDTA. Tumor-infiltrating immune cells were separated from other cells by centrifugation with the deceleration brake set at 1 at 1,260 g at 4oC for 25 minutes in a Ficoll gradient (17-1440-03, GE Healthcare). After centrifugation, TILs were collected for staining for flow cytometry. For in vitro T cell-coculture experiments, Splenic CD4+ or CD8+ T cells were isolated from naïve WT C57BL/6J mice using MojoSort Mouse CD4 T cell isolation Kit or MojoSort Mouse CD8 T cell isolation Kit, respectively and activated using Dynabeads Mouse T-Activator CD3/CD28 (GIBCO, Cat# 11452D) in RPMI medium plus 10% FBS for 16 hr before adding to tumor cells at a tumor-to-T-cell (E:T) ratio of 1:2 for 48 hr. Cells were processed and stained for flow cytometry.
Instrument	BD LSR Fortessa, 17 Parameters
Software	FlowJo X and GraphPad Prizm (v. 9.3.0).
Cell population abundance	The abundance is depended on the specific population, from 0-80%.
Gating strategy	The gating strategy for each experiment was supplied in supplementary figures. They were determined by single color stains where applicable. Generally, the first gating (FCS/SCC) was done on alive cells to exclude debris and dead cells, followed by the selection of single cells (FCS-H/SSC-W & FCS-H/FSC-W), excluding clumps. Then the specific fluorophores were confirmed

and analyzed by comparing single color stains.

☒ Tick this box to confirm that a figure exemplifying the gating strategy is provided in the Supplementary Information.
